# Evolving Perspectives on Patient-Accessible Electronic Health Records in Sweden: Comparative Survey Study

**DOI:** 10.2196/76757

**Published:** 2026-11-13

**Authors:** Jonas Moll, Hanife Rexhepi, Gunilla Myreteg, Charlotte Blease, Annika Bärkås, Åsa Cajander, Josefin Hagström, Isto Huvila, Maria Hägglund, Bridget Kane, Gunnar O Klein, Sari Kujala, Irene Muli, Rose-Mharie Åhlfeldt, Anna Kharko

**Affiliations:** 1 Department of Informatics, Örebro University School of Business, Örebro University, Novahuset, 4th floor, Fakultetsgatan 1, Örebro, 701 82, Sweden, 46 19 303878; 2 Division of Computer and System Sciences, School of Informatics, University of Skövde, Skövde, Sweden; 3 Department of Business Studies, Uppsala University, Uppsala, Sweden; 4 Department of Women's and Children's Health, Uppsala University, Uppsala, Sweden; 5 Division of General Medicine, Department of Medicine, Beth Israel Deaconess Medical Center, Harvard Medical School, Boston, MA, United States; 6 Department of Information Technology, Uppsala University, Uppsala, Sweden; 7 Department of ALM, Uppsala University, Uppsala, Sweden; 8 Medtech Science and Innovation Centre, Uppsala University Hospital, Uppsala, Sweden; 9 Business School, Karlstad University, Karlstad, Sweden; 10 Department of Computer Science, Aalto University, Espoo, Finland; 11 Centre for Primary Care and Health Services Research, University of Manchester, Manchester, United Kingdom

**Keywords:** patient-accessible electronic health records, PAEHRs, patient engagement, eHealth, comparative study, health care communication, ORA, open notes, online record access, national survey

## Abstract

**Background:**

Patient-accessible electronic health records (PAEHRs) aim to enhance patient engagement by providing access to clinical information. In Sweden, implementation of a national PAEHR began in 2012 and reached nationwide scale-up in 2018. Adoption and use have steadily increased over time. So far, few studies report on how perspectives on PAEHRs evolve, informing on long-term use and development of the services.

**Objective:**

Data were compared from 2 national patient surveys conducted in Sweden (in 2016 and 2022) regarding patients’ experiences and usage of PAEHRs. The aim is to explore and understand possible changes in patients’ reasons for use and perceived value of the different information types and functions provided.

**Methods:**

The 2016 survey included responses from 2587 respondents, while the 2022 survey included 13,008 respondents. In 2016, the proportion of respondents who identified as women was 66.8% (1629/2437) and 67.3% (8753/13,008) in 2022. The largest age groups in 2016 were 55‐64 years and 65‐74 years in 2022. The surveys collected data on patients’ experiences with PAEHRs, including demographic information, reasons for using, and perceived impacts on communication with health care professionals. Users who logged in to the PAEHR during the survey periods were invited to participate in the respective survey through a link on the login page.

**Results:**

Chi-square tests (*P*<.05) were used to check for differences in responses between the 2 surveys. Due to the large difference in the number of respondents between the 2 surveys, the effect sizes are fairly small, ranging from 0.02 to 0.19. Key findings indicate an increase in the positive perception of PAEHRs over the past 6 years. In 2022, 72.3% of respondents reported that PAEHRs supported communication with health care professionals, compared with 60.0% in 2016 (*P*<.001). The primary reason for using PAEHRs shifted from general interest in 2016 to preparing for health care visits in 2022. There was also an increase in patients valuing access to their lab test results, from 95.0% in 2016 to 97.8% in 2022 (*P*<.001; Cramer V=0.06).

**Conclusions:**

The study demonstrates that the overall highly positive patient attitude towards and usage of PAEHRs in 2016 persisted and was strengthened in 2022. The findings point to the growing importance of PAEHRs in enhancing patient engagement and communication with health care professionals, and to the acceptance and promotion of PAEHR within the Swedish health care system. This research provides valuable insights into the evolving landscape of PAEHR and its impact on patient-centered care. Increased use of PAEHRs may suggest that such systems can support better-informed patients, improve transparency in health care, and facilitate patient participation in care. The results can guide future policies and the development of PAEHR worldwide.

## Introduction

Internationally, the implementation of patient-accessible electronic health records (PAEHRs) has become increasingly widespread [1,2], though full access is still far from the norm. Some countries, such as Norway [3], Finland [4], and Estonia [5], offer nationwide PAEHR services that provide most adult citizens with access to their health information. Similar initiatives have been initiated in the United States, where the OpenNotes movement has made access to clinical notes a legal right [6]. In Sweden, PAEHRs were first introduced in 2012 in Region Uppsala. Today, all residents except adolescents aged 13 to 16 years can access their electronic health records (EHRs) through the national PAEHR service Journalen, available via the 1177 eHealth portal [7]. As of January 2025, more than 8 million individuals have accessed Journalen at least once since its launch, with 1.8 million users in January 2025 alone. During the same month, the service recorded over 7.5 million logins. Women currently represent approximately 53% of users, while men account for 47% [8].

Journalen provides patients with access to clinical information (eg, clinical notes, laboratory test results, medications, diagnoses, referrals, treatment plans, vaccinations). However, the availability of specific information still varies by region and health care provider. This variation stems from Sweden’s decentralized health care system, which consists of 21 self-governing regions and multiple private providers operating across regional boundaries. Each region maintains its own policies and regulations regarding which data are made available to patients through Journalen. As a result, the type of information accessible to patients still differs depending on the region in which they have received care.

Studies show that PAEHRs increase patient activation and engagement, and strengthen patient-clinician relationships [9]. The growing body of evidence on patients’ PAEHR shows that shared clinical notes support patients in preparing for upcoming appointments, improve communication between clinicians and patients, and can actively engage patients in their health care [10-14]. Despite the growing evidence of their benefits for patients, health care professionals remain doubtful, believing that many patients risk getting confused or anxious if they read their clinical notes [15], especially regarding mental health conditions [16]. Some professionals fear this will lead to a rise in patient contact and greater workplace burdens [17]. However, despite PAEHR being argued to cause these burdens and concerns, it has not been empirically confirmed [18,19]. Considering the rapidly increasing number of users, it is important to understand patients’ and health care professionals’ needs and requirements to find common ground without compromising the patient’s autonomy or the professionals’ expertise. A systematic review regarding the impacts of PAEHRs shows that many studies highlight the positive outcomes and that fewer studies report concerns (security, privacy, and confidentiality issues, and the possible risk of anxiety for some patients) [20].

Furthermore, a growing body of research reveals that most patients want unrestricted access to their medical records [21-23]. Health care in Sweden, the Nordic countries, and The OpenNotes Collaborative in the US have established the immediate release of clinical notes as best practice [21]. However, the practice of immediately releasing laboratory test results - without the clinician having first informed the patient - remains controversial. In a US study [24], out of 8139 respondents, 96% (7520/7859) of patients preferred receiving immediate access to test results even if their health care practitioner had not yet performed a review. This preference also applied to patients getting informed of abnormal results. Some patients (411/5473, 7.5%) experienced increased worry, especially when test results were abnormal. Similar results have been reported in Sweden [2,23].

There is a lack of research on changes in long-term PAEHR usage. Previous research reports snapshot images of use and attitudes or outcomes at a single point in time. In contrast, this study applies a longitudinal approach by analyzing survey data collected within the same national context at 2 time points—2016 and 2022. The aim is to explore and better understand potential shifts in patients’ experiences and use of PAEHRs, their perceived value of access to different types of information, the impact of PAEHR on communication with health care professionals, and technical changes related to the types of information made available. By comparing developments over time, this study offers insights into how such eHealth initiatives have evolved. This knowledge could be useful for governmental organizations and health care providers in their ongoing quest to develop the PAEHR (its functions and information types) and how to address complex issues, such as finding a balance between general (national) needs and local variations.

## Methods

### Study Context

In 2016, the first Swedish national survey on patients using their PAEHR was conducted [2]. At that time, 17 of the 21 regions offered web-based access to clinical notes, while only 7 provided access to laboratory test results (Figure 1). The results revealed highly positive attitudes toward PAEHR among responding patients [2].

**Figure 1.**
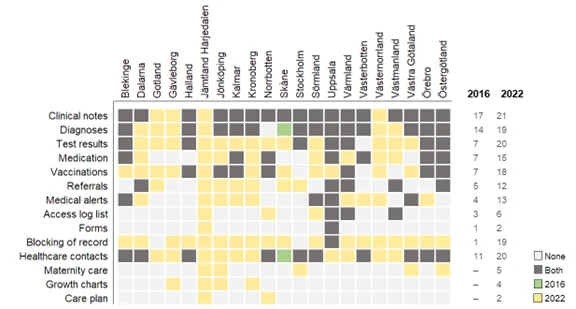
Availability of various information in 1177 Journalen in each region of Sweden in 2016 and 2022.

Much has changed since 2016. In 2022, six years after the first survey study, a second extensive patient survey was conducted; this time as part of the Nordic research initiative NORDeHEALTH [25]. All 21 regions provided extensive access to Journalen’s content (Figure 1). For instance, all regions allowed access to clinical notes, and 20 of 21 to laboratory results. Whereas in 2016, only half of the regions provided an overview of health care contacts, by 2022, all but one had implemented this feature. Another significant change was that all but 2 regions enabled patients to restrict access to parts of their medical records from other caregivers. Information on vaccinations was available in 18 regions (up from 7 in 2016), on medications in 15 regions (up from 7), and on referrals in 12 regions (up from 5). Since Journalen’s launch in 2012, the system has evolved substantially [8]. The number of users has increased, the minimum age for youth access has been lowered from 18 to 16 years, and patients now have access to a broader range of health information.

### Participant Characteristics, Inclusion, and Exclusion

The target population consisted of users who used the Swedish PAEHR and who accessed Journalen during the data collection period in 2016 and 2022. The samples included respondents across different age groups, genders, and educational backgrounds (Table 1).

**Table 1.** Demographic characteristics of survey respondents, N (%) in 2016 and 2022 surveys regarding gender, age, and education.

Demographic	2016	2022
Gender, n (%)^a^		
Women	1629 (66.8)	8754 (67.3)
Men	798 (32.7)	4177 (32.1)
Other	10 (0.4)	77 (0.6)
Age (years), n (%)^b^		
15‐19	16 (0.7)	218 (1.7)
20‐24	126 (5.1)	409 (3.1)
25‐34	400 (16.3)	1735 (13.3)
35‐44	363 (14.8)	1867 (14.4)
45‐54	457 (18.7)	2274 (17.5)
55‐64	484 (19.8)	2539 (19.5)
65‐74	449 (18.3)	2614 (20.1)
75‐84	131 (5.3)	1262 (9.7)
85 or older	24 (1.0)	90 (0.7)
Education, n (%)^c^		
No formal education	66 (2.8)	75 (0.6)
Primary education	159 (6.7)	1106 (8.5)
Upper secondary education	656 (27.7)	3434 (26.4)
Higher education	1410 (59.6)	8015 (61.6)
Research education	75 (3.2)	377 (2.9)

a For gender, n=2437 in 2016 and n=13,008 in 2022.

b For age, n=2450 in 2016 and n=13,008 in 2022.

c For education, n=2366 in 2016 and n=13,008 in 2022.

The minimum age for participants differed between the surveys. In 2016, participants were required to be at least 18 years old, and in 2022, the minimum age was 15 years. The differences reflect the changes in the rules of access for adolescents. No other inclusion criteria were applied.

### Sampling Procedures

To recruit respondents, both surveys were advertised in the section of the national patient portal in Sweden, 1177 (1177.se) that hosts the PAEHR Journalen. To view the survey advertisement, users were required to log into their PAEHR account, ensuring that only verified and active users had the opportunity to participate. There were some differences in survey distribution strategies regarding the lowest allowed age of survey participants and the length of the data collection period (Table 2).

**Table 2.** Overview of survey distribution strategies, age requirements, and participants’ response metrics for the 2016 and 2022 patient-accessible electronic health record (PAEHR) studies.

Characteristic		2016 survey	2022 survey
Data collection			
Survey platform		Webropol	Webropol
Time period		Jun 2016-Oct 2016	Jan 2022-Feb 2022
Participants			
Minimum age requirement (years)		18	15
Verified active users, n		423,141	1,085,092
Completed survey, n (%)		2489 (0.59)	13,008 (1.20)

The verified active users (Table 2) are approximations, based on openly available monthly data of unique users of all ages [8]. The minimum age for participation was unrestricted in 2016 and limited to 15 years in 2022 (age of own consent). The age of resident access, online record access, differed: in 2016 access was given from 18 years of age, and in 2022 from 16 years of age (possibly from 13 years, after application).

### Sample Size, Power, and Precision

In total, 2498 respondents completed the survey in 2016 and 13,008 in 2022. These results correspond to participation rates of 0.59% and 1.20%, respectively (Table 2). The effect sizes in the statistical comparison were relatively small.

### Measures and Covariates

Both surveys collected data on different aspects of respondents’ use and experiences of PAEHRs, as well as sociodemographic characteristics. Age, gender, and education were also included as covariates in the analysis. More specifically, the surveys from 2016 and 2022 consisted of single/multiple-choice and open questions covering several themes. In the 2016 survey, the themes were: (1) General questions related to the eHealth service Journalen; (2) Experiences from accessing and using the content of Journalen; (3) Information security; (4) Information needs, behavior, and information-seeking style; (5) Personal health; (6) Demographics. In the NORDeHEALTH 2022 Patient Survey, the themes were similar: (1) Sociodemographic information; (2) Experience with health care; (3) Experience with PAEHR through the patient portal 1177.se; (4) Reasons for and impact of using the health record; (5) Errors, omissions, and offenses; (6) Security and privacy; and (7) Usefulness of information and functions. Original surveys are presented in Multimedia Appendices 1 and 2.

The 2016 survey was developed from earlier studies examining patients’ and health care professionals’ perspectives on PAEHRs in Sweden [2,15,17]. It focused on key areas identified as important for understanding user experiences and attitudes toward PAEHRs. The 2022 survey was based on the 2016 version but was further developed using insights from similar studies conducted in Finland, Norway, and the United States [21,26,27], and covered areas that required further exploration. Before distribution, the 2022 survey was piloted to ensure clarity, relevance, and comparability.

In both surveys, the questions were a mixture of single-choice and multiple-choice questions. Both surveys had questions asking the respondent to provide a single rating on a 5-point agreement Likert scale. Key aspects in survey design are summarized in Table 3.

**Table 3.** Overview of survey structure and question types in 2016 and 2022 surveys.

Characteristic	2016 survey	2022 survey
Survey structure		
Language	Swedish	Swedish
Closed/choice questions	Optional	Mandatory
Open questions	Optional	Mandatory
Whole survey, n (%)	128 (100)	83 (100)
Closed/choice questions	128 (100)	76 (92)
Open questions	0	7 (8)
Questions used in this analysis, n (%)		
Closed/choice questions	27 (100)	28 (100)

### Conditions and Design

The study compares survey responses from 2 time periods, 2016 and 2022, from the same national context.

### Data Diagnostics

Before the data analysis, some variables were adjusted to make the 2 surveys comparable. Age in the 2016 survey, originally reported as birth year, was coded into the interval categories used in 2022. Some categories related to education in the 2016 survey were also coded to the categories of the 2022 study. Minor differences in question wording were also reviewed and adjusted.

Since some data were missing in the 2016 study (all participants did not answer all questions), the proportions of missing data per question were calculated for each of the demographic variables used in the study (age, gender, and education). This was not done for the 2022 study, where all participants answered all questions.

### Analytic Strategy

Aside from descriptive analysis, chi-square tests were used to uncover groupwise differences between the 2 surveys. The significance level was preset to 0.05 for all tests. Effect sizes were calculated using Cramer’s V.

Regarding encouragement by health care to read in Journalen, the questions in the 2 surveys were asked differently, and no statistical test was performed. Only descriptive data on the proportion of patients who had been encouraged to read by someone working in health care were considered here.

Chi-square tests were performed to detect differences between the 2 surveys with regard to why respondents chose to read in Journalen. Again, only a descriptive analysis was performed of the items that were related but not directly comparable: “To follow up what has been said during a health care visit” in the 2016 survey versus “To remember the care plan/follow my treatment recommendations” and “To be sure I understood what the doctor said” in the 2022 survey.

Additionally, the investigation of the perceived usefulness of information and functions in Journalen differed between the 2 surveys, preventing direct comparisons. For example, the responses to the question “How important is it for you to have access to the following information which is wholly or partly based on information contained in ‘Journalen’” in 2016 and to the statement “It would be useful for me to have access to the following information or functions” in 2022 could not be directly compared with a chi-square test. The response alternatives “Disagree” to “Agree” were, however, the same. Since both versions measured participants’ perceived utility of the various types of information and functions, a decision was made to combine “Disagree” and “Partly disagree” into “Not valuable” and “Agree” and “Partly agree” into “Valuable.” The neutral option was labeled “Neither.” The chi-square test was then used to check for possible statistically significant associations between surveys and the perceived value of the different information types and functions.

Last, responses to the information and function items were compared between the 2016 survey and a matched stratified subsample of the 2022 survey. We applied the stratified random sampling with proportional allocation [28]. The 2022 subsample was matched by age and gender, with the size of each bin (eg, women aged 15‐19 years) dictated by the 2016 data. Since there were more 2022 survey data, data points were randomly selected. Comparative analysis was carried out using the chi-square test as previously described.

### Ethical Considerations

Both surveys acquired ethical approval prior to data collection. The 2016 survey was approved by the Regional Ethical Review Board in Uppsala, Sweden (EPN 2016/129). The NORDeHEALTH 2022 Patient Survey received ethical authorization from the appropriate governing body in all countries where it was distributed, in Sweden from the Ethical Review Authority (Approval #2021‐05229). Prior to participation in the 2016 and 2022 surveys, all respondents were required to provide informed consent, confirming their voluntary involvement in the study. Thus, participation was voluntary and was not monetarily incentivized or compensated. All responses were collected anonymously, and no identifiable personal data were stored or processed; hence, no identification of individual respondents can be done based on the content of the manuscript or supplementary material.

## Results

### General Characteristics

Table 1 provides demographic information about the respondents to the different surveys. In the 2016 survey, 2587 respondents completed the questionnaire, while in 2022, the number increased to 13,008. These numbers correspond to participation rates of 0.59% and 1.20%, respectively.

In 2016, 66.8% (1629/2437) identified as women, and in 2022, this proportion was 67.3% (8754/13,008). In both survey rounds, the proportion of female respondents exceeded the proportion of female users of 1177 Journalen according to usage statistics, which indicated that 54% of users in 2022 were women [8]. In the 2016 survey, the largest age group among respondents was 55‐64 years (484 out of 2450, 19.8%), whereas in 2022, the largest group shifted to those aged 65‐74 years (2614 out of 13,008, 20.1%).

Among the respondents of the 2016 survey, the most common education level was higher education (1410/2366, 59.6%), followed by upper secondary education (656/2366, 27.7%). A similar result pattern can be seen in the 2022 survey with 61.6% (8015/13,008) of the respondents having a higher education and 26.4% (3434/13,008) having an upper secondary education. Only 2.8% (866/2366) of the respondents from 2016 and 0.6% (75/13,008) from 2022 had no formal education. In the 2016 group, the overall health among the respondents was good (1123/2415, 46.5%), while a slightly higher proportion of the respondents from the 2022 survey reported their health to be neutral (4992/12,686, 39.4% versus 444/2415, 18.4% in 2016), thus neither good nor bad. Overall, the distributions over genders, ages, and educational levels in the different surveys were fairly similar.

### Encouragement to Use PAEHR

Both surveys included a question regarding the extent to which health care professionals encouraged patients to read the information available in Journalen. In 2022, 16% (2035/13,008) of respondents reported having been encouraged compared with 8% (203/2458) in 2016. It is, however, not possible to specify the contexts in which the patient received this advice, or concerning the type of information the patient was encouraged to read.

### The Impact of PAEHR on Communication

Table 4 summarizes the responses to whether access to Journalen improved communication between respondents and health care professionals. In both survey years, the majority of participants reported experiencing enhanced communication. Notably, a significantly higher proportion of respondents in 2022 perceived Journalen as helpful in facilitating communication compared with 2016 *χ*^2^_2_=166.46 (N=15,435), *P*<.001.

**Table 4.** Impact of patient-accessible electronic health record (PAEHR) on communication between respondents and health care professionals in 2016 and 2022 surveys. In 2016, the survey item was “Information in *Journalen* has helped me in communication with medical staff” and in 2022 “Journalen supports communication between myself and healthcare professionals.”

Survey	Helpful, n (%)	Neutral, n (%)	Not helpful, n (%)
2016 (n=2427)	1455 (60.0)	667 (27.5)	305 (12.6)
2022 (n=13,008)	9409 (72.3)	2247 (17.3)	1352 (10.4)

### Reasons for Using PAEHR

As shown in Table 5, the majority of the respondents in both surveys agreed that they used Journalen out of general interest/curiosity, to get an overview of their medical history and treatment, and to prepare for health care visits.

**Table 5.** Reasons for using patient-accessible electronic health record (PAEHR) in 2016 and 2022 surveys, based on the survey question: “Please indicate how much you disagree or agree with the following statements. I read my health record online”.

Survey item^a^		2016	2022	Chi-square (*df*)	*P* value	Effect size
“Mostly general interest” (2016) / “Out of general curiosity” (2022), n (%)^b^				95.52 (1)	<.001	0.08
Agree		1553 (64.0)	6924 (53.2)			
Neither		449 (18.5)	2297 (17.7)			
Do not agree		423 (17.4)	3787 (29.1)			
“To get an overview of my medical history and treatment” (2016/2022), n (%)^c^				426.5 (1)	<.001	0.16
Agree		2331 (92.8)	9597 (73.8)			
Neither		99 (3.9)	1864 (14.3)			
Do not agree		83 (3.3)	1547 (11.9)			
“Because I am not sure if I got the right care” (2016/2022), n (%)^d^				281.7 (1)	<.001	0.13
Agree		731 (30.1)	2061 (15.8)			
Neither		564 (23.2)	1950 (15.0)			
Do not agree		1131 (46.6)	8997 (69.2)			
“Because I suspect inaccuracies” (2016/2022), n (%)^e^				33.57 (1)	<.001	0.05
Agree		512 (21.1)	2114 (16.3)			
Neither		534 (22.0)	1808 (13.9)			
Do not agree		1385 (57.0)	9086 (69.8)			
“To prepare for my health care visit” (2016) / “To prepare myself for a consultation or hospitalization” (2022) , n (%)^f^				16.74 (1)	<.001	0.03
Agree		1401 (57.0)	7992 (61.4)			
Neither		504 (20.5)	2029 (15.6)			
Do not agree		551 (22.4)	2987 (23.0)			

a Items had different wording in the surveys (Multimedia Appendices 1 and 2).

b n=2425 for 2016 and n=13,008 for 2022.

c n=2513 for 2016 and n=13,008 for 2022.

d n=2426 for 2016 and n=13,008 for 2022.

e n=2431 for 2016 and n=13,008 for 2022.

f n=2456 for 2016 and n=13,008 for 2022.

The most popular reason for accessing Journalen among respondents in both surveys was to obtain an overview of their medical history and treatment. However, the proportion was significantly lower in 2022 (2331/2513, 92.8% vs 9597/13,008, 73.8%; *P*<.001). The second most popular reason for accessing Journalen differed between the 2 surveys. In 2016, the second most popular reason was general interest, while in 2022, it shifted to preparing for a consultation or hospitalization. The reason that ranked second in 2016 dropped in 2022, indicating that although general interest and curiosity remained a strong motivating factor for patients, there was a shift towards using the PAEHR for more specific information needs related to their health and health care.

In both surveys, questions regarding inaccuracies and uncertainty about the quality of received care were considered to be relatively unimportant reasons for accessing Journalen. However, the proportion of respondents who agreed that they used Journalen for these reasons was significantly smaller in the 2022 survey compared with that in 2016 (*P*<.001).

Another reason for patients’ use of Journalen is to review and follow up on the discussions and agreements made during meetings or consultations with health care professionals. The 2 surveys asked about this topic using different wordings, meaning the responses are not directly comparable. Table 6 presents the results related to using Journalen to follow up on what was discussed during a health care encounter.

**Table 6.** Reasons for using the patient-accessible electronic health record (PAEHR), comparing similar items in 2016 and 2022.

Survey item	Agree, n (%)	Neither, n (%)	Do not agree, n (%)
To follow up what has been said during a health care visit (2016), n=2512	2221 (88.4)	146 (5.8)	145 (5.8)
To remember the care plan/follow my treatment recommendations (2022), n=13,008	9667 (74.3)	1578 (12.1)	1763 (13.6)
To be sure I understood what the doctor said (2022), n=13,008	6605 (50.8)	2321 (17.8)	4082 (31.4)

In the 2016 survey, the vast majority of the respondents (2221/2512, 88.4%) used Journalen to follow up on what was said during a health care encounter. In the 2022 survey, this question was split into two reasons: (1) to ensure understanding of the information conveyed during the visit, and (2) to help remember and follow the care plan and treatment recommendations. The results from 2022 indicate that a large majority of the respondents (9667/13,008, 74.3%) used Journalen for helping to remember the care plan or treatment recommendations, and around half of the respondents (6605/13,008, 50.8%) used Journalen for ensuring that they understood what the doctor had said.

### Perceived Value of PAEHR Information and Functions

Regarding the perceived value of different information types available in Journalen, several significant differences were identified between the surveys (Table 7). Having access to test results was highly valued by patients in both surveys. The rating is slightly higher in the 2022 survey (12,720/13,008, 97.8%) compared with 2016 (2332/2455, 95.0%). The importance of having access to information regarding vaccinations (12,268/13,008, 94.3%) and referrals (12,179/13,008, 93.6%) handled by health care has also increased (compared with 80.4% [1957/2434] and 89.9% [2199/2445], respectively, in 2016). The largest increase could be seen for vaccinations. All these increases were significant (*P*<.001). The data trends persisted when stratified survey samples were compared (Figure 2).

**Table 7.** Perceived value of patient-accessible electronic health record (PAEHR) information in 2016 and 2022 surveys, based on the survey questions: “How important is it for you to have access to the following information which is wholly or partly based on information contained in ‘Journalen’ (2016) / It would be useful for me to have access to the following information or functions (2022)”.

Information^a^		2016	2022	Chi-square (*df*)	*P* value	Effect size
Referrals (content and how it is handled in care) (2016/2022), n (%)^b^				43.26 (1)	<.001	0.05
Valuable		2199 (89.9)	12,179 (93.6)			
Neither		199 (8.1)	628 (4.8)			
Not valuable		47 (1.9)	201 (1.5)			
List of all pharmaceuticals (2016 /2022), n (%)^c^				32.87 (1)	<.001	0.05
Valuable		2197 (89.5)	12,082 (92.9)			
Neither		186 (7.6)	629 (4.8)			
Not valuable		71 (2.9)	297 (2.3)			
Overview of all vaccinations (2016) / Overview of vaccinations (2022), n (%)^d^				546.30 (1)	<.001	0.19
Valuable		1957 (80.4)	12,268 (94.3)			
Neither		366 (15.0)	540 (4.2)			
Not valuable		11 (4.6)	200 (1.5)			
Results of tests (2016 / 2022), n (%)^e^				62.41 (1)	<.001	0.06
Valuable		2332 (95.0)	12,720 (97.8)			
Neither		84 (3.4)	210 (1.6)			
Not valuable		39 (1.6)	78 (0.6)			
Overview of all health care contacts (2016/2022), n (%)^f^				18.52 (1)	<.001	0.04
Valuable		2209 (90.6)	12,112 (93.1)			
Neither		172 (7.1)	723 (5.6)			
Not valuable		56 (2.3)	173 (1.3)			

a Items had different wording in the surveys (Multimedia Appendices 1 and 2).

b n=2445 for 2016 and n=13,008 for 2022.

c n=2454 for 2016 and n=13,008 for 2022.

d n=2434 for 2016 and n=13,008 for 2022.

e n=2455 for 2016 and n=13,008 for 2022.

f n=2437 for 2016 and n=13,008 for 2022.

**Figure 2.**
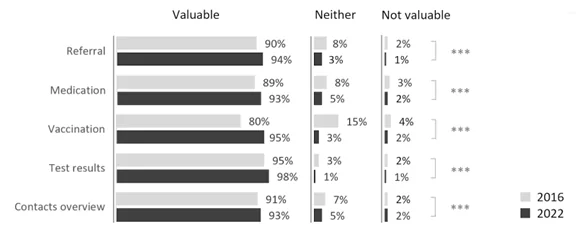
Comparison of information items using a subsample of responses from 2016 (n=2118) and 2022 (n=2118) stratified by gender and age. Significance levels indicate results of chi-square testing, where ***P<.001, **P<.01, *P<.05. Note that items had different wording in the surveys (Multimedia Appendices 1 and 2).

A similar trend, where respondents in 2022 rated certain functions as more valuable than in 2016 (Table 8), was also observed in relation to the ability to contribute personal information and data to the medical record. For example, a higher proportion of respondents in 2022 considered the option to upload health declaration forms prior to visits as useful (10,976 out of 13,008, 84.4%), compared with 2016 (1801 out of 2421, 74.4%). The same trend was evident for the possibility of contributing information from self-testing or home monitoring. In this case, 67.2% (8747/13,008) of the respondents found this possibility useful in 2022 compared with 53.4% (1287/2410) in 2016. In both these cases, the increase in the proportions of respondents that found these functions useful was statistically significant (*P*<.001). The data trends persisted when stratified survey samples were compared (Figure 3).

**Table 8.** Perceived value of patient-accessible electronic health record (PAEHR) functions in 2016 and 2022 surveys, based on the survey questions: “How important is it for you to have access to the following information which is wholly or partly based on information contained in ‘Journalen’ (2016) / It would be useful for me to have access to the following information or functions (2022).”

Function^a^		2016	2022	Chi-square (*df*)	*P* value	Effect size
Ability to order and manage medical certificate and other certificates (2016/2022), n (%)^b^				17.11 (1)	<.001	0.03
Valuable		1927 (79.6)	10,802 (83.0)			
Neither		370 (15.3)	1475 (11.3)			
Not valuable		125 (5.2)	731 (5.6)			
Ability to point out errors I find in the record (2016/2022), n (%)^c^				6.32 (1)	.012	0.02
Valuable		1981 (82.0)	10,929 (84.0)			
Neither		295 (12.2)	1522 (11.7)			
Not valuable		141 (5.8)	557 (4.3)			
Ability to write comments to text in “Journalen” (2016) / Ability to write own comments to text in the health record (2022), n (%)^d^				5.78 (1)	.016	0.02
Valuable		1254 (51.8)	6389 (49.1)			
Neither		598 (24.7)	3177 (24.4)			
Not valuable		570 (23.5)	3442 (26.5)			
Contribute with information on health, for example by providing Health declaration for next visit (2016/2022), n (%)^e^				143.10 (1)	<.001	0.10
Valuable		1801 (74.4)	10,976 (84.4)			
Neither		453 (18.7)	1472 (11.3)			
Not valuable		167 (6.9)	560 (4.3)			
Contribute with information of self-testing/monitoring at home (2016/2022), n (%)^f^				171.4 (1)	<.001	0.11
Valuable		1287 (53.4)	8747 (67.2)			
Neither		704 (29.2)	2952 (22.7)			
Not valuable		419 (17.4)	1309 (10.1)			
Contribute information about expectations for the health care visit (2016/2022), n (%)^g^				0.11 (1)	.73	—^h^
Valuable		1305 (54.3)	7013 (53.9)			
Neither		687 (28.6)	3894 (29.9)			
Not valuable		412 (17.1)	2101 (16.2)			
Ability to contact health care electronically and ask questions about medical record (2016/2022), n (%)^i^				9.94 (1)	.002	0.03
Valuable		1860 (76.9)	10,374 (79.8)			
Neither		344 (14.2)	1710 (31.1)			
Not valuable		214 (8.9)	924 (7.1)			
Ability to block certain medical records from access by other medical staff (2016/2022), n (%)^j^				65.06 (1)	<.001	0.07
Valuable		964 (40.1)	4121 (31.7)			
Neither		693 (28.8)	4060 (31.2)			
Not valuable		747 (31.1)	4827 (37.1)			
See which care units and staff groups have been inside “Journalen” (see log data) (2016) / See which care units and staff groups have accessed my information (see log data) (2022), n (%)^k^				24.13 (1)	<.001	0.04
Valuable		1796 (74.3)	9022 (69.4)			
Neither		353 (14.6)	2160 (16.6)			
Not valuable		267 (11.1)	1826 (14.0)			
Ability to access information and manage services for my children (2016 / 2022), n (%)^l^				3.65 (1)	.06	0.02
Valuable		1166 (49.9)	6769 (52.0)			
Neither		660 (28.2)	2861 (22.0)			
Not valuable		511 (21.9)	3378 (26.0)			

a Items had different wording in the surveys, see Multimedia Appendices 1 and 2.

b n=2422 for 2016 and n=13,008 for 2022.

c n=2417 for 2016 and n=13,008 for 2022.

d n=2422 for 2016 and n=13,008 for 2022.

e n=2421 for 2016 and n=13,008 for 2022.

f n=2410 for 2016 and n=13,008 for 2022.

g n=2404 for 2016 and n=13,008 for 2022.

h Not available.

i n=2418 for 2016 and n=13,008 for 2022.

j n=2404 for 2016 and n=13,008 for 2022.

k n=2416 for 2016 and n=13,008 for 2022.

l n=2337 for 2016 and n=13,008 for 2022.

**Figure 3.**
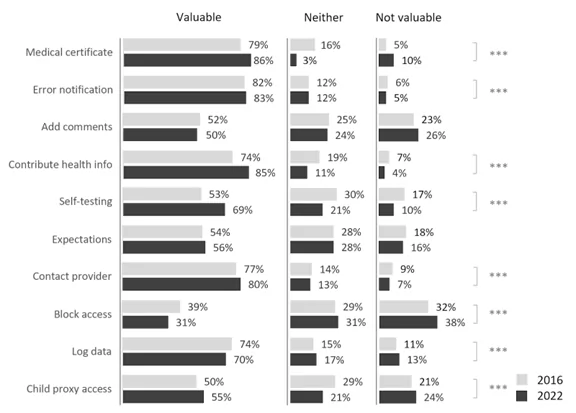
Comparison of function items using a subsample of responses from 2016 (n=2118) and 2022 (n=2118) stratified by gender and age. Significance levels indicate results of chi-square testing, where ***P<.001, **P<.01, *P<.05. Note that items had different wording in the surveys, (Multimedia Appendices 1 and 2).

There were also instances in which participants in the 2022 survey reported assigning less value to certain types of information or functions in Journalen compared with participants in the 2016 survey. The perceived value of information regarding who has accessed the patient’s medical record (presented in the “log list,” which displays the name, role, department, and date of access) has slightly decreased in the 2022 survey (9022 out of 13,008, 69.4%) compared with the 2016 survey (1796 out of 2416, 74.3%).

A similar trend was observed regarding the option to block (parts of) the medical record from access by other health care professionals. In 2022, 31.7% (4121 out of 13,008) considered the blocking feature important, compared with 40.1% of respondents (964 out of 2404) in the 2016 survey. In both these cases, the analysis showed that the increase in the proportions of respondents who did not find these functions to be useful was statistically significant (*P*<.001). It is of interest to note that despite the relatively low and decreasing values for the possibility to block, blocking is one of the items that in 2022 was offered to patients in all but 2 regions, while it was only offered in one region in 2016 (Figure 1).

## Discussion

### Principal Findings

This study is the first of its kind in Sweden, and one of the few internationally, to focus on how user experiences with PAEHR evolve over time. We identified several noteworthy differences between the respondents of our 2016 survey and those who participated in 2022, both in terms of their experiences and their usage of the PAEHR Journalen. While using PAEHR out of general interest to have an overview of one’s health has slightly declined, the use of Journalen to prepare for consultations or upcoming hospitalizations has increased, indicating PAEHR is turning to an ordinary information source rather than one consulted out of curiosity. Moreover, patients increasingly reported being encouraged by health care professionals to access the PAEHR, and more users found that reading their medical record helps in communication with health care. Nonetheless, some patients still did not find it helpful—although this number decreases.

The perceived value of various information types and functions also changed. Respondents reported increased perceived value of features such as access to test results, vaccination records, referral information, and the ability to upload personal health data. In contrast, the perceived value of features like the option to block parts of the record has declined.

### Changes in Reasons for Accessing the PAEHR

The most popular reason for respondents to access Journalen is to get an overview of their medical history and treatment. In 2016, the second most popular reason for using Journalen was out of general interest, while in 2022 it was to prepare for the upcoming visits, while the reading because of interest/curiosity was pushed down to third place. These results support and expand the findings from earlier studies. The growing evidence base on PAEHRs shows that patients benefit from reading their clinical notes [29,30]. This study shows that the continuing main reasons for accessing Journalen, as also suggested by earlier research conducted in different contexts during these years, are to obtain an overview of one’s medical history and treatment, and to prepare for the next visit [2,10,31].

In both the 2016 and 2022 surveys, a low proportion of respondents were accessing Journalen due to suspicions of inaccuracies in the documentation. The proportion in the 2022 survey was significantly lower in comparison to that in 2016. This stands in contrast to concerns expressed by health care professionals, who have been worried about increased workload due to telephone contact caused by documentation inaccuracies or ambiguities found by patients [18], as well as worries about being “checked” by patients [17]. It is also important to highlight that suspicions of inaccuracies do not necessarily indicate that patients doubt the information. Instead, it may reflect a desire to verify that the information is accurate. Our interpretation is that respondents in the 2022 survey are less doubtful about the PAEHR, which could be a consequence of repeated access to and review of their records without encountering major errors or facing consequences of inaccuracies. As patients become more acquainted with reading their medical information and gain a better understanding of the language and documentation practices used by health care professionals, it is reasonable to expect that uncertainties and doubts may decrease over time.

Furthermore, the finding that usage in 2022 was more future-oriented, in that higher proportions of respondents used Journalen for preparations and managing self-care, and less focused on reviewing the doctor’s notes is given health care professionals’ concerns that patients might struggle to understand the content presented in Journalen [15]. Patients’ main self-reported reasons for reading the PAEHR were not related to mitigating problems of understanding. Instead, their main reasons for using Journalen include obtaining an overview, curiosity/interest, preparing for appointments, and managing their own care and treatment.

### Changes to Effects on Communication and Encouragement to Read

Patients report that reading clinical notes brings them substantial benefits. The respondents from both the 2016 and 2022 surveys reported that reading and reviewing their medical information was helpful for communication with health care. A statistically significantly higher proportion of users perceived that Journalen has been helpful in communication with health care professionals in 2022 than in 2016. A possible reason for this could be that the PAEHR has been established and is now more recognized as a potential communication aid by patients and health care professionals.

Improved communication between patients and health care professionals, following patients’ review of their medical information, has been reported in earlier research [13, 14]. This study clearly indicates that respondents not only see PAEHRs as a communication aid, but also suggest that the benefits of access in this regard have become more pronounced over time. One possible interpretation is that the broader range of information available in 2022 compared with 2016 (Figure 1) contributed to this development. It is reasonable to assume that access to more comprehensive information empowers patients to ask more informed questions and better understand what is communicated during consultations [32]. Furthermore, it is interesting to note that there is a group of respondents (although reducing in number) who did not find Journalen to be helpful for facilitating communication (12.6% in the 2016 survey and 10.6% in the 2022 survey). A tentative explanation could be that we here find the youngest and the oldest survey participants, who because of their age, might experience lower digital health literacy, which would undermine communication-related benefits [33].

Health care professionals are increasingly encouraging patients to access Journalen. As shown in this study, the proportion of patients who reported being encouraged to use Journalen doubled from 8% in 2016 to 16% in 2022. A hopeful interpretation of this trend is that health care professionals may be moving away from initial concerns about increased patient anxiety or additional workload. This interpretation is supported by the growing number of patients who report receiving such encouragement from their care providers. Earlier research highlighted a more hesitant stance among physicians. For instance, doctors often framed the medical record as “their work tool” [15], not something intended for patient access, and typically did not recommend that patients read it [17]. In contrast, the current findings suggest a shift toward a more positive and supportive attitude among health care professionals regarding patient access to medical records. This trend can be viewed through the lens of prior research examining how technologies become embedded and legitimized in health care settings. The increasing recommendation of Journalen may reflect growing acceptance and normalization of patient digital tools in clinical practice [34]. The findings of this study suggest that the Swedish PAEHR is increasingly viewed as a legitimate and valuable tool not only by patients but also by health care professionals. In comparison, elsewhere in the Nordics, the proportion of patients who are encouraged remains higher. In a Finnish survey of patients with chronic diseases, 30% of the respondents reported that health care professionals had encouraged them to use eHealth services, 70% of which concerned the national PAEHR, My Kanta [35]. In 2022, 43.8% of the respondents in a national population survey reported being referred to use OmaKanta [36]. Those patients who were not referred to use OmaKanta were less likely to use the service. The reason may be that the implementation of the Finnish national PAEHR started already in 2010 [37].

### Changes to the Perceived Value of Information Content and Functions

Considering the percentage of patients who valued being able to access their test results in 2016 and 2022, almost every patient highly valued having such access, which is in line with earlier research [24]. Other functions also showed an increase in perceived value. These included access to information about vaccinations and referrals, as well as the ability for patients to upload their own health data. We found limited prior research specifically addressing patient evaluations of these particular information types. In the case of vaccination information, our interpretation is that the COVID-19 pandemic played a significant role. The pandemic heightened the need for patients to keep track of their vaccination history and to monitor whether additional doses were needed, thereby increasing the perceived value of this feature. Moreover, the increasing possibility for patients in different regions to access these types of information (Figure 1) may also affect their evaluations of its value. A diffusion of PAEHR functions over the country may increase patients’ need and usage of that type of information. Contradicting this interpretation, however, is the evaluation of the possibility to block parts of the medical record, which showed less value in 2022, despite the fact that the function was by then available in all regions but two. Our interpretation is that this function is not generally needed by any type of patient, but is restricted to specific groups of patients. For example, a patient who was treated for a mental health condition might want to block this part of the EHR so that it is not visible to somatic health providers [38,39]. Previous research has also highlighted other types of sensitive information that some patients may prefer to keep private, such as records related to domestic violence, genetic data, sexual health, substance use, and reproductive health [40]. Therefore, while the overall perceived value of this function may be low, it remains crucial for a smaller subset of patients who may consider it highly important.

Furthermore, the function that allows patients to see who has accessed their EHR (the log list) was not available in all regions (being offered in 3 regions in 2016 and 6 regions in 2022). Responses indicate a lower reported value of this function over time. However, this finding should be interpreted with caution, given its limited and uneven availability. Our interpretation is that this type of information is not generally needed by most patients, but may be highly valued by individuals with particularly sensitive medical records. This interpretation is supported by previous research [40]. Additionally, the log list itself may be difficult for patients to interpret, as it typically consists of names that they may not recognize or associate with specific care events. It is also worth noting that many respondents in the 2016 survey had current or previous experience working in health care. This may have contributed to the initially higher interest in the log list, an interest that may not reflect the broader patient population.

In summary, the evaluation of different information types and functions reveals both increasing and decreasing values over time. Changes in perceived value may be explained by several factors: shifts in the external environment (such as the COVID-19 pandemic), increased access enhancing the perceived usefulness of certain features, the need for a specific function is limited to smaller groups of patients, or a decline in interest once a function is realized to be less useful in practice.

### Implications

The findings show a notable demographic shift toward older users of Journalen in 2022, which suggests that eHealth services are being adopted by a broader population of patients over time. Older persons, who have been traditionally less engaged with digital technologies, appear to be increasingly engaged in using PAEHRs. Previous research indicates that older patients, and in some cases their relatives through proxy access, particularly value the possibility to review medical information and prepare for treatment-related discussions at their own pace [41]. This may help explain the growing engagement among older users. The increased use observed in Sweden may also have been influenced by the COVID-19 pandemic, which heightened the need for remote access to health information and increased overall familiarity with digital health services.

The increase in the percentage of patients being encouraged to use Journalen by health care professionals from 2016 to 2022 reflects a growing acceptance and endorsement of PAEHRs within the health care system. This encouragement likely contributes to the higher adoption rates observed. Since the number of patients that are recommended to use Journalen is still low in 2022 (around 16% of respondents), interventions to promote health care professionals’ encouragement could still be recommended. When health care providers actively promote the use of PAEHRs, it signals to patients that these tools are valuable and trustworthy resources for managing their health. This endorsement can significantly influence patient behavior, leading to greater engagement with their medical records and more proactive health management [35]. The active role of health care professionals in promoting PAEHRs also helps address potential concerns and barriers patients might have, such as fears about privacy or the complexity of using digital tools.

The consistently high value placed on accessing test results underscores the importance of transparency and the immediate availability of medical information for patients. This type of information could be seen as convenient to access through a PAEHR, since additional communication with health care is often not required. Ensuring that these features are maintained and enhanced will continue to be critical for patient satisfaction, as well as patient participation. Test results are a vital component of clinical information that patients rely on to understand their health status and make informed decisions about their care. Immediate access to these results helps patients track their health metrics, prepare for follow-up consultations, and engage in discussions with their health care professionals about their treatment plans [23]. Transparency in providing test results can also reduce patient anxiety by eliminating delays and uncertainties associated with waiting for results. Maintaining high standards of transparency and accessibility to such information in PAEHRs to meet patients’ expectations can help to foster a sense of control and empowerment in managing their health.

### Strengths and Limitations

This study is based on the largest surveys conducted in Sweden on patients’ views about PAEHR since the eHealth service was first implemented. However, the study has some limitations relating to the use of a non-probability sample and a limited sample size. Also, the participation rate was relatively low, at 0.6% in 2016 and 1.2% in 2022. Moreover, possible respondents were limited to those who used Journalen during the administration of the survey, and actual respondents were those who volunteered to participate. It is, therefore, not possible to infer that our samples were representative of the opinions of patients in Sweden. The sample was, however, stratified according to gender and age. The large difference in the number of respondents in the 2 surveys also resulted in low effect sizes in the statistical comparisons made. In the stratified subsample analyses, however, the significances remained, indicating robustness in the findings. Responder biases, such as acquiescence or prior enthusiasm or skepticism about the topic, may have influenced the decision to take part in the survey, potentially impacting the findings. The missing data analysis, presented in Multimedia Appendix 3, shows that somewhat more men than women have missing responses. There is also a similar trend for older respondents and respondents with lower education. The proportions of missing data are, however, in most cases small.

The larger number of respondents in 2022 should not be interpreted as indicating that the sample was more representative of the patient population than in 2016. Instead, this increase may reflect broader changes, such as greater encouragement to use PAEHRs, increased system maturity, improved usability, and expanded availability of information and functions over time. These developments are likely to have lowered barriers to use and encouraged participation among a wider range of patients. Respondents’ level of education as well as their current health status might also have impacted the willingness to participate in the surveys. Most respondents reported their health to be “good” or “neutral,” which suggests that the less healthy patients might have refrained from participation in the survey.

Last, respondents were somewhat older in the 2022 survey. It is not possible to verify whether this is representative of the overall users. It might be that older patients are also more frequent users of PAEHR and therefore happened to log in during the time that the survey was open. The high participation rate among older patients could be seen as a positive sign, suggesting that they are both willing and able to engage with digital health services.

### Conclusions and Future Research

By comparing national survey data from 2016 and 2022, this study highlights significant improvements in patient experiences with PAEHRs in Sweden. In 2022, a greater proportion of patients reported that PAEHRs supported communication with health care professionals. This suggests that, over time, as PAEHRs become more integrated into patients’ everyday health information practices, their potential to enhance patient-provider communication can be realized more fully.

A clear indication of a demographic shift towards older users suggests a broader adoption of PAEHRs over time. The doubling in health care professionals encouraging patients to use Journalen, from 8% in 2016 to 16% in 2022, reflects the growing acceptance and promotion of PAEHRs within the health care system. Although there is an increase in encouragement, there are still relatively low levels of professionals who seem to encourage patients to use PAEHRs. This may highlight a need for integrating PAEHR promotion into routine clinical practice.

Additionally, the high-value patients placed on accessing test results remained consistent, underscoring the importance of transparency and immediate availability of health information. These trends are not unique to the Swedish health care system. International research points in the same direction, indicating a global move toward greater integration and normalization of PAEHRs [42]. Furthermore, comparisons between the 2 surveys show that respondents reported a high interest in accessing test results and information about their care, and many perceived that access supported communication. These findings highlight the positive impact of PAEHRs on promoting patient engagement in their own care and improving communication among health care professionals.

In general, the findings from this study demonstrate a shift in how patients engage with their health information and where access and digital interaction become an integrated part of their care. As PAHERs continue to evolve and become a more natural part of the clinical practice, their role may expand beyond access to information to actively shape how patients participate and influence their health care.

Further research is needed, as many questions remain unanswered. For instance, while this study indicates a general support in communication, it does not provide insights into the specific contexts or patient groups for whom PAEHR use does not enhance communication. Additionally, the survey data does not clarify in which situations, for which types of patients, concerning what kinds of information, or from which health care professionals, patients are encouraged to access Journalen. These are important factors to explore in order to better understand how PAEHRs can be effectively integrated into care and tailored to support diverse patient needs.

Communication practices and health care professionals’ encouragement for patients to engage with their medical information could be further explored through the conceptualization of legitimacy as a social infrastructure [34]. This perspective gives an opportunity to more closely study the process of acceptance of PAEHRs with a practical and action-oriented focus. Such studies could develop further understandings of similarities and differences between local and national requirements, needs, and the status of PAEHR.

The study presented in this paper is unique in that no similar comparative study that follows user experiences over time has been performed in Sweden before. Similar international studies are very few to date. The results show an increase in the use of PAEHRs and may suggest that such eHealth systems can support better-informed patients, improve transparency in health care, and facilitate patient participation in care. The results may therefore inform future policy decisions and the continued development of PAEHRs in Sweden and beyond.

## Supplementary material

10.2196/76757 Multimedia Appendix 1 The 2022 survey.

10.2196/76757 Multimedia Appendix 2 The 2016 survey.

10.2196/76757 Multimedia Appendix 3 Missing data analysis.

## Abbreviations

EHR: electronic health record
PAEHR: patient-accessible electronic health record
