# Supplementary material for: Evolving Perspectives on Patient-Accessible Electronic Health Records in Sweden: Comparative Survey Study
Source: J Med Internet Res. 2026 Nov 13;28:e76757. doi: 10.2196/76757 (PMC13766677; doi:10.2196/76757)
Supplement: Supplementary file 1 [file jmir-v28-e76757-s001.docx]

**General Background information**

**1) In the last 2 years, have you received care from a doctor or other health professional for (mark the relevant options, several marks are permitted)?**

- Mental health condition(s)
- Cancer care
- Other health problem(s)
- No treatment

If mental health OR cancer is “yes”: **Have you read the health information (your journal) from your mental health care OR cancer treatment online?**

- I read all/almost all of my record
- I read some of the record
- I did not read the record

If mental health conditions is “yes”: **When you received mental health care, at what level did it take place? (mark the relevant options, several marks are permitted. This is a voluntary questions, voluntary for the country)**

- primary care
- hospital outpatient (specialist consultation)
- hospitalized/inpatient care

If mental health conditions is “yes”: **For how long have you received mental health care?**

- less than 3 months
- 3 months – 1 year
- 1-3 years
- over 3 years

**General questions about use**

**2) Did any of the following encourage you to read your health record? [check all that apply].**

- Health professionals

o Physician

o Nurse

o Psychologist

o Physiotherapist

o Other medical staff

o Don’t remember who

- Written information provided by the hospital / healthcare facility /clinic
- Family or friends
- Web-pages, as the National health portal web-page, etc.
- Newspapers, radio, TV, Facebook, etc.
- Other: ______________________

**3) How often did you access your health record during the last 12 months?**

- This is my first time
- 1-3 times
- 4-9 times
- More than 10 times

**Experience with accessing the health record**

**4) Please indicate how much you disagree or agree with the following statements (Likert scale 1-5 disagree-agree).**

**I read my health record online:**

- out of general curiosity
- to improve my understanding about my health issue
- to prepare myself for a consultation or hospitalization
- to get an overview of my medical history and/or treatment
- to be sure I understood what the doctor said
- to remember the care plan/follow my treatment recommendations
- because I suspect inaccuracies
- to share documents with relatives or friends
- to share documents with health professionals who do not have access
- because I am not sure if I got the right care
- Other - please specify (free text)

**5) Please indicate how much you disagree or agree with the following statements (Likert scale 1-5 disagree-agree).**

**It is important for me to be able to share my record**

- with relatives and friends
- with health professionals who do not have access

**6) Please indicate how much you agree or disagree with the following statements (Likert scale 1-5 disagree-agree).**

**Having access to my health record**

- helps me trust my health care provider more
- supports communication between myself and healthcare professionals

**7) Have you ever found anything in your record you thought was a mistake (not counting misspellings or typographical errors)?**

- Yes, I found one or more mistakes
- No, I didn’t find any mistakes
- Don’t know/don’t remember

**IF YES TO Q7) How important was the most serious mistake you found?**

- Not at all serious
- Somewhat serious
- Very serious
- I am not sure

**IF YES TO Q7). Please describe the most serious mistake. (don’t include names, identifying information or sensitive information)** We will not report this (or any information in this survey) to your providers. If you are concerned about a possible mistake please discuss it with your providers.

FREE TEXT!

**IF YES to Q7) Did you do any of the following when you found a mistake in your record? (choose the most important one)**

- Informed the health professional at the next visit
- Contacted the health care unit via phone.
- Did nothing
- Something else (free text)

**8) How confident do you feel in your ability to find mistakes in your record?**

- Not at all confident
- A little confident
- Confident
- Very confident

**9) Have you ever felt offended by something you read?**

- Yes
- No

IF YES Please explain

**Documents/information and functionalities of available services in the health portal (country specific)**

**10) Please rate the usefulness of the following documents/information or functions available online. If you have not read one of the documents, please select “Not read”.**

**(Not read; Not useful at all; Not very useful; Neutral; Useful; Very useful; Do not know)**

**(here will all the documents from the next question, that your patients have access to, follow)**

**Documents/information:**

- Content of referrals
- Referral status
- List of all pharmaceuticals / my medications
- Overview of vaccinations
- Results of tests (not available in all regions)
- Clinical notes from primary care
- Clinical notes from hospital care
- Overview of all health care contacts

**Functions:**

- The core/summary record with critical information (in Finland: Risk information + Diagnosis list)
- Ability to access information and manage services for my children (not available in all regions)
- Ability to access information and manage services for my family members (In Finland and in Estonia)
- See which care units and staff groups have accessed my information (see log data)
- Ability to block certain medical records from access by other medical staff
- Organ donation consent (in Finland, Estonia and Norway)
- Living will (in Finland)
- Ability to contact health care provider electronically and ask questions about medical record
- Ability to communicate electronically with other patients
- Ability to point out errors I find in the health record
- Ability to write own comments to text in the health record
- Contribute with information on health, for example by providing Health declaration for next visit
- Contribute with information of self-testing/monitoring at home
- Contribute information about expectations for the health care visit
- Ability to order and manage sick leave medical certificates and other certificates (vaccinations, etc.)

**11) It would be useful for me to have access to the following information or functions (country specific)**

**(this is information/documents that might be offered through several different services, available through the national health portals (or similar))**

**Documents/information:**

- Referrals (content and how it is handled in care)
- List of all pharmaceuticals / my medications
- Overview of vaccinations
- Results of tests (not available in all regions)
- Clinical notes from primary care
- Clinical notes from hospital care
- Overview of all health care contacts

**Functions:**

- The core/summary record with critical information (in Finland: Risk information + Diagnosis list)
- Ability to access information and manage services for my children (not available in all regions)
- Ability to access information and manage services for my family members (In Finland)
- See which care units and staff groups have accessed my information (see log data)
- Ability to block certain medical records from access by other medical staff
- Organ donation consent (in Finland and Norway)
- Living will (in Finland)
- Ability to contact health care provider electronically and ask questions about medical record
- Ability to communicate electronically with other patients
- Ability to point out errors I find in the health record
- Ability to write own comments to text in the health record
- Contribute with information on health, for example by providing Health declaration for next visit
- Contribute with information of self-testing/monitoring at home
- Contribute information about expectations for the health care visit
- Ability to order and manage medical certificate and other certificates

**Information Security and Privacy**

**12) What is your opinion on information security and privacy?**

**Please indicate how much you disagree or agree with the following statements (Likert scale 1-5 disagree-agree).**

- I think that my health record generally maintains a high level of security.
- I trust that only authorized medical staff are accessing my health records.
- Patients should be able to see who has accessed their patient information.
- When I log in to the health record, I trust the log-in process.
- I have no privacy concerns with being able to enter additional health information from my own sources in the health record, such as exercise data, EKG-data and other health data from private applications.
- I have no privacy concerns with copying my health information from the health record to other online applications (example: Google Health, Apple Health, Facebook etc).
- I would like to manage who should have access to my health record (for example that a certain diagnosis is not accessible to certain groups of staff or relatives).

**13) How often do you copy and paste information from your health records to another online application (Google Health, Apple Health, Facebook etc)?**

- I have never shared information
- I have shared information a few times (1-4 times)
- I have shared information frequently (more than 5 times)

**14) Have you experienced that family, friends or another have demanded access to health records that you did not want to share?**

- Yes \ No \ I don’t know

IF YES: Who demanded access to health records that you did not want to share?

- a family member
- a friend
- other: _______________________

**15) Have you experienced that someone has seen health records that you did not want to share?**

- Yes \ No \ I don’t know

IF YES: Who accessed your record without your consent?

- a healthcare professional
- a family member
- a friend
- other: _______________________

**16) Do you consider some types of health information especially sensitive?**

- Yes / No

IF YES; can you give an example of what type of health information that is most sensitive to you? ____________________________ (free text)

**C Do you think that all your health information is equally sensitive? (add if relevant)**

- Yes / No

IF NO, can you give examples of what type of health information that is most sensitive to you? ____________________________ (free text)

**Usability**

**17) Please evaluate your experience of the system.**

Scale: 1 (Strongly disagree) to 7 (Strongly agree), 8 (I don’t know)**:**

1. The system meets my needs.

2. Using this system is a frustrating experience.

3. The system is easy to use.

**18) Have you had a very positive experience with the system?**

- Yes/No

IF YES, please describe the positive experience here as clearly as possible:

**19) Have you had a very negative experience with the system?**

- Yes/No

IF YES, please describe the very negative experience here as clearly as possible:

**Multidisciplinary Teams (MDT / MDK)**

Multidisciplinary teams are sometimes used to manage particular diseases, and will consist of different specialities, depending on the disease being managed.

**20) Do you know if your care has been discussed at a multidisciplinary team (MDT) meeting?**

- Yes / No / Don’t know

IF YES please answer the following (Q20a, Q20b and Q20c)

**20a**: Were you invited to the MDT meeting when your care was discussed?

- Yes / No

**20b:** What was your experience of the MDT?

- (Free text)

**20c:** Do you have access to documentation from the MDT meeting?

- Yes / No / Don’t know

**Background characteristics**

**21) Region/county:** Specified by each country

**22) Gender:**

- Female
- Male
- Other

**23) Age:**

- 16 - 24 years
- 25 - 34 years
- 35 - 44 years
- 45 - 54 years
- 55 - 64 years
- 65 - 74 years
- 75 – 84 years
- 85 years or more

**24) Highest completed education: (Country specific)**

- Research, (third cycle) of higher education
- Higher education, >3 years (second cycle-master)
- Higher education <= 3 years (first cycle -bachelor)
- Higher vocational education ( vocational diploma)
- 12 years school -Upper secondary education
- Elementary school
- No formal education

**25) Do you have a health professional education?**

- Yes
- No

**26) Which of the following best describes your current employment status? Choose the one that is most relevant.**

- Full time
- Part time
- Student
- Retired
- Unemployed
- Not able to work
- None of the above (free text)

**27) How is your overall health?**

- Very bad
- Bad
- Fair
- Good
- Very Good
- I don’t know / I don’t want to answer
