# Supplementary material for: Evolving Perspectives on Patient-Accessible Electronic Health Records in Sweden: Comparative Survey Study
Source: J Med Internet Res. 2026 Nov 13;28:e76757. doi: 10.2196/76757 (PMC13766677; doi:10.2196/76757)
Supplement: Supplementary file 2 [file jmir-v28-e76757-s002.docx]

Questionnaire - Patient empowerment - Evaluation of the patient's online medical records

How well does the content of the following issues in:

## General Questions on “*Journalen*”

# 1. I received information about the possibility to use “*Journalen*” via:

Medical professionals	☐
Media	☐
Information on the care unit	☐
Friends / Relatives	☐

Other (specify) _____________________________________________________

# 2. How often do you use “*Journalen*”?

Several times a day	☐
Once a week	☐
Several times a week	☐
Once a month	☐

# 3. What is your attitude towards “*Journalen*”?

Strongly	Disagree	Neutral	Agree	Strongly

disagree	agree

I believe that access to medical records online is generally
a good reform	☐	☐	☐	☐	☐

I believe that access to "Journalen" is good for me

☐	☐	☐	☐	☐

I would consider to change health care providers to get one
that gives me access to ”Journalen”	☐	☐	☐	☐	☐

4. **Why do you use “*Journalen*”?**

Strongly	Disagree	Neutral	Agree	Strongly

disagree	agree

Mostly general interest	☐	☐	☐	☐	☐

To get an overview of my
medical history and treatment	☐	☐	☐	☐	☐

To get an overview of my relatives’
medical history and treatment	☐	☐	☐	☐	☐

Because I am not sure if I got the right care	☐	☐	☐	☐	☐

To follow up what has been said during a health care visit	☐	☐	☐	☐	☐

Because I suspect inaccuracies	☐	☐	☐	☐	☐

To prepare for my health care visit	☐	☐	☐	☐	☐

To become more involved in my care	☐	☐	☐	☐	☐

Other (specify) _____________________________________________________

# 5. How important is it for you to be able to access patient information?

Strongly	Disagree	Neutral	Agree	Strongly

disagree	agree

It improves communication between medical staff and me	☐	☐	☐	☐	☐

It leads to improvements in health and social care	☐	☐	☐	☐	☐

It improves the understanding of the condition	☐	☐	☐	☐	☐

It makes me feel safe	☐	☐	☐	☐	☐

It makes me feel informed	☐	☐	☐	☐	☐

It leads to that I can take care of my health better	☐	☐	☐	☐	☐

It leads to that I can take care of my relatives health better	☐	☐	☐	☐	☐

It is essential that I am able to actively participate in decisions
about my or my relatives’ health	☐	☐	☐	☐	☐

For my own documentation	☐	☐	☐	☐	☐

It has no relevance	☐	☐	☐	☐	☐

Other (specify) _____________________________________________________

# 6. How long do you think is reasonable to have to wait after a health care visit before you have access to your medical records via “Journalen”?

Same day (unsigned / uncertified information)	☐

After a day (unsigned / uncertified information)	☐

Two weeks (can be both signed / certified
 and unsigned / uncertified data)	☐

One month (can be both signed / certified
 and unsigned / uncertified data)	☐

Other (specify) **_____________________________________________________**

# 7. To what extend do you agree with the following statements regarding your relationship with health care?

Strongly	Disagree	Neutral	Agree	Strongly

disagree	agree

To take part of the patient information via “Journalen”
has affected the relationship with health care system positively	☐	☐	☐	☐	☐

Medical staff has informed me about the possibility to
read “*Journalen*”	☐	☐	☐	☐	☐

Medical staff has encouraged me to use the „*Journalen*”	☐	☐	☐	☐	☐

I discuss the content of “*Journalen*” with medical staff	☐	☐	☐	☐	☐

### 8. What do you think about the usability of „*Journalen*”?

Strongly	Disagree	Neutral	Agree	Strongly

disagree	agree

I think I want to use “Journalen” regularly	☐	☐	☐	☐	☐

I think that “*Journalen*” is more complicated than
it needs to be	☐	☐	☐	☐	☐

I think that “*Journalen*” is easy to use	☐	☐	☐	☐	☐

I think I would need personal technical support
to use “*Journalen*”	☐	☐	☐	☐	☐

I think that the various functions in “*Journalen*” work
well together	☐	☐	☐	☐	☐

I think there are many elements in “*Journalen*” that
are not consistent	☐	☐	☐	☐	☐

I think that most people could learn to use “*Journalen*”
fairly quickly	☐	☐	☐	☐	☐

I think that “*Journalen*” is difficult to use	☐	☐	☐	☐	☐

I feel very safe and secure (about what I do)
 when I use “*Journalen*”	☐	☐	☐	☐	☐

I need to learn quite a lot before I can start
 using “*Journalen*”	☐	☐	☐	☐	☐

I as a user of 1177.se find it difficult to find the link to “*Journalen*”	☐	☐	☐	☐	☐

Other (specify) _____________________________________________________

# Information Content

# 9. How accurate are the following statements regarding your understanding of the content in “*Journalen*”?

Strongly	Disagree	Neutral	Agree	Strongly

disagree	agree

I understand most of what is in the medical records	☐	☐	☐	☐	☐

I think that the medical records contain too much technical language	☐	☐	☐	☐	☐

I think that the medical records should be written

more comprehensible	☐	☐	☐	☐	☐

I understand most of the test results	☐	☐	☐	☐	☐

I understand most of the log list	☐	☐	☐	☐	☐

I understand most of the referral function	☐	☐	☐	☐	☐

# 10. What would you do if you see something in “*Journalen*” you do not understand?

Strongly	Disagree	Neutral	Agree	Strongly

disagree	agree

Contact the current health care unit via phone	☐	☐	☐	☐	☐

Ask medical staff at the next visit	☐	☐	☐	☐	☐

Ask a medically trained person, e.g. via phone on 1177	☐	☐	☐	☐	☐

Ask an anonymous question via www.1177.se	☐	☐	☐	☐	☐

Ask someone who I know personally,
in family or among acquaintances	☐	☐	☐	☐	☐

Look for information myself, e.g. via Internet	☐	☐	☐	☐	☐

Use social media, e.g. discussion forums	☐	☐	☐	☐	☐

Do nothing	☐	☐	☐	☐	☐

Other (specify) **_____________________________________________________**

# 11. How do you share information in “*Journalen*” with others?

I use the Share-function in “*Journalen*”
to appoint representatives and share information	☐	☐	☐	☐	☐

Discuss it with relative	☐	☐	☐	☐	☐

Discuss it with medical staff	☐	☐	☐	☐	☐

Other (specify) _____________________________________________________

If you use the Share-function in “*Journalen*”, how do you share the information?

I share all information in “*Journalen*”	☐

I share only selected parts in “*Journalen*”	☐

# 12. Have you read anything in “*Journalen*” that made you feel anxious?

Yes	☐

No	☐

**If yes, what did you do?**

Contacted health care via phone	☐

Waited until the next visit	☐

Contacted a friend with medical background	☐

Contacted patient association / patient committee	☐

Tried to find information about it on the Internet	☐

None	☐

Other (specify) _____________________________________________________

13. **Have you read anything in “*Journalen*” that has made you feel upset?**

Yes	☐

No	☐

If you like, specify why you felt upset: **______________________________**

**If yes, what did you do?**

Contacted health care via phone	☐

Waited until the next visit	☐

Contacted a friend who works in health care	☐

Contacted patient association / patient committee	☐

Wrote about it on social media	☐

None	☐

Other (specify) _____________________________________________________

# 14. Have you read anything in “*Journalen*” that was wrong, such as wrong information and / or data?

Yes	☐

No	☐

Do not know	☐

**If yes, what did you do?**

Contacted health care via phone	☐

Waited until the next visit	☐

Contacted a friend with medical background	☐

Contacted patient association / patient committee	☐

Tried to find information about it on the Internet	☐

None	☐

Other (specify) _____________________________________________________

# 15. How accurate is the content of “*Journalen*”?

Strongly	Disagree	Neutral	Agree	Strongly

disagree	agree

The content in the record reflects the information I think
that health care has about me	☐	☐	☐	☐	☐

There is information about me that is missing in the record which I think
should be there and that the staff should know	☐	☐	☐	☐	☐

If you consider that there is information missing that should be there, specify what type of information is missing: _______________________________________________________________________________________________

# 16. How important is “*Journalen*” to make you feel that you are involved in your own care?

Strongly	Disagree	Neutral	Agree	Strongly

disagree	agree

Information in “*Journalen*” has helped me
in communication with medical staff	☐	☐	☐	☐	☐

Information in “*Journalen*” had a positive impact on the
ability to work together with medical staff
making decisions about care and treatment	☐	☐	☐	☐	☐

Information in “*Journalen*” had a positive impact on the
 ability to follow the prescription of treatment	☐	☐	☐	☐	☐

Information in “*Journalen*” had a positive impact on the
ability to take own steps to improve health	☐	☐	☐	☐	☐

Other (specify) _____________________________________________________

# 17. How important is it for you to have access the following information which is wholly or partly based on information contained in “*Journalen*”

Strongly	Disagree	Neutral	Agree	Strongly

disagree	agree

Referral (content and how it is handled in care)	☐	☐	☐	☐	☐

List of all pharmaceuticals	☐	☐	☐	☐	☐

Overview of all vaccinations	☐	☐	☐	☐	☐

Results of tests	☐	☐	☐	☐	☐

Overview of all health care contacts	☐	☐	☐	☐	☐

Being able to read record entries from psychiatry	☐	☐	☐	☐	☐

Being able to read all types of record entries	☐	☐	☐	☐	☐

Ability to order and manage medical certificate and other certificates	☐	☐	☐	☐	☐

Ability to point out errors I find in the record	☐	☐	☐	☐	☐

Ability to write own comments to text in “*Journalen*”	☐	☐	☐	☐	☐

Contribute with information on health, for example by providing

Health declaration for next visit	☐	☐	☐	☐	☐

Contribute with information of self-testing/monitoring at home	☐	☐	☐	☐	☐

Contribute information about expectations for the health care visit	☐	☐	☐	☐	☐

Ability to contact health care electronically and ask questions about
medical record	☐	☐	☐	☐	☐

Ability to communicate electronically with other patients	☐	☐	☐	☐	☐

Ability to block certain medical records from access by other
medical staff	☐	☐	☐	☐	☐

See which care units and staff groups have been inside “*Journalen*”
(see log data)

☐	☐	☐	☐	☐

Ability to access information and manage services for my children	☐	☐	☐	☐	☐

Other (specify) _____________________________________________________

# Security

# 18. How do you perceive the level of security in “*Journalen*”?

Strongly	Disagree	Neutral	Agree	Strongly

disagree	agree

As far as I can judge, I think that “*Journalen*” generally

Maintains a high level of security.	☐	☐	☐	☐	☐

I trust that only authorized medical staff is accessing my
medical records in “*Journalen*”.	☐	☐	☐	☐	☐

It is good that I as a patient am able to take part in the log list
and see who has accessed my patient data	☐	☐	☐	☐	☐

Other (specify) _____________________________________________________

# General questions on information

# 19. How do you receive bad news about your health from your health care provider?

By Phone	☐

In connection with a health care visit	☐
Via mail	☐

Via other “web / eServices”	☐

Other (specify) _____________________________________________________

# 20. How would you want to receive bad news about your health?

By Phone	☐

In connection with a health care visit	☐
Via mail	☐

In connection with that I read alone about it in “*Journalen*”	☐

Other (specify) _____________________________________________________

# 21. How accurate are the following statements regarding access to health information?

Strongly	Disagree	Neutral	Agree	Strongly

disagree	agree

It is important (for me) to get information about health	☐	☐	☐	☐	☐

I want to get health information from many different sources	☐	☐	☐	☐	☐

I compare the health information I have received from various sources	☐	☐	☐	☐	☐

It is easy to determine in what situations I need health information	☐	☐	☐	☐	☐

I know where I can get health information	☐	☐	☐	☐	☐

I can use the health information I have received or acquired

in order to take care of my own or other people's health	☐	☐	☐	☐	☐

I easily get hold of the health information I need

a) in printed sources (magazines, books)	☐	☐	☐	☐	☐

b) on the internet	☐	☐	☐	☐	☐

c) on TV or radio	☐	☐	☐	☐	☐

d) from medical staff (e.g. doctors, nurses)	☐	☐	☐	☐	☐

e) from other people	☐	☐	☐	☐	☐

f) from health care information system etc.	☐	☐	☐	☐	☐

It is difficult to know whom to trust regarding

health issues/questions	☐	☐	☐	☐	☐

It is easy to determine whether the health information I received from the following sources are reliable or not

a) in printed sources (magazines, books)	☐	☐	☐	☐	☐

b) on the internet	☐	☐	☐	☐	☐

c) on TV or radio	☐	☐	☐	☐	☐

d) from medical staff (e.g. doctors, nurses)	☐	☐	☐	☐	☐

e) from other people	☐	☐	☐	☐	☐

It is difficult to understand words or sentences used in
 health information	☐	☐	☐	☐	☐

It is easy to understand the drugs package inserts, labels or
 prescription	☐	☐	☐	☐	☐

I get way too much information about health	☐	☐	☐	☐	☐

I do not want to think about matters related to health	☐	☐	☐	☐	☐

I have avoided to read, listen to or watch health information

a) in printed sources (magazines, books)	☐	☐	☐	☐	☐

b) on the internet	☐	☐	☐	☐	☐

c) on TV or radio	☐	☐	☐	☐	☐

d) from medical staff (e.g. doctors, nurses)	☐	☐	☐	☐	☐

e) from other people	☐	☐	☐	☐	☐

The health information I receive

a) helps me often to understand or learn something new	☐	☐	☐	☐	☐

b) often confirms my previous understanding	☐	☐	☐	☐	☐

c) often conflict with my previous views	☐	☐	☐	☐	☐

I can affect my own health.	☐	☐	☐	☐	☐

# 22. What other sources do you use to find health information?

www.1177.se	☐

Internetmedicin.se	☐

Netdoktor.se	☐

Health magazines	☐

Books	☐

Peer-to-peer sources, e.g. discussion forums, Facebook)	☐

Other (specify) _____________________________________________________

# Questions about your health

# 23. I am part of one of the following disease groupings:

Cancer	☐

Mental health	☐

Diabetic	☐

High blood pressure	☐

Other (specify) _____________________________________________________

# 24. I consider myself

Strongly	Disagree	Neutral	Agree	Strongly

disagree	agree

My health is very good	☐	☐	☐	☐	☐

I am very worried about my health	☐	☐	☐	☐	☐

I often think about my health	☐	☐	☐	☐	☐

# Demographic Questions

**Age**

Year of Birth: _______

**Gender**

Female	☐

Male	☐

Other	☐

**Do you work (or have you worked) in health care?**

Yes	☐

No	☐

**Education? (Select the highest education / degree you have received / completed)**

No formal education	☐

Compulsory education (eg Elementary school,
junior secondary school)	☐

High school, less than 3 years	☐

High school, 3 years or more	☐

Higher education, less than 3 years	☐

Higher education, 3 years or more	☐

Doctorate	☐

Other (specify) _____________________________________________________

**I belong to the following county**

Blekinge county council	☐

Dalarna county council	☐

Region Gotland	☐

Region Gävleborg	☐

Region Halland	☐

Region Jämtland/Härjedalen	☐

Region Jönköpings län	☐

Kalmar county council	☐

Kronoberg county council	☐

Norrbotten county council	☐

Region Skåne	☐

Stockholm county council	☐

Sörmland county council	☐

Region Uppsala	☐

Värmland county council	☐

Västerbottens county council	☐

Västernorrland county council	☐

Västmanland county council	☐

Region Västra Götaland	☐

Region Örebro	☐

Region Östergötland	☐

**I have also received health care in another county council**

Blekinge county council	☐

Dalarna county council	☐

Region Gotland	☐

Region Gävleborg	☐

Region Halland	☐

Region Jämtland/Härjedalen	☐

Region Jönköpings län	☐

Kalmar county council	☐

Kronoberg county council	☐

Norrbotten county council	☐

Region Skåne	☐

Stockholm county council	☐

Sörmland county council	☐

Region Uppsala	☐

Värmland county council	☐

Västerbottens county council	☐

Västernorrland county council	☐

Västmanland county council	☐

Region Västra Götaland	☐

Region Örebro	☐

Region Östergötland	☐
