# Supplementary material for: Evolving Perspectives on Patient-Accessible Electronic Health Records in Sweden: Comparative Survey Study
Source: J Med Internet Res. 2026 Nov 13;28:e76757. doi: 10.2196/76757 (PMC13766677; doi:10.2196/76757)
Supplement: Supplementary file 3 [file jmir-v28-e76757-s003.docx]

Table S1. Missing data analysis with regards to gender and survey questions about communication with medical staff and reasons for use.

|  | **Women** | **Men** | **Other** |
| --- | --- | --- | --- |
| **Information in Journalen has helped me in communication with medical staff**, n(%) | 19 (1.2%) | 13 (1.6%) | 0 (0%) |
| **I read my health record online mostly out of general interest**, n(%) | 68 (4.2%) | 67 (8.4%) | 0 (0%) |
| **I read my health record online to get an overview of my medical history and treatment**, n(%) | 30 (1.8%) | 20 (2.5%) | 0 (0%) |
| **I read my health record online because I am not sure if I got the right care**, n(%) | 80 (4.9%) | 53 (6.6%) | 0 (0%) |
| **I read my health record online because I suspect inaccuracies**, n(%) | 74 (4.5%) | 55 (6.9%) | 0 (0%) |
| **I read my health record online to prepare for my health care visit**, n(%) | 60 (3.7%) | 42 (5.3%) | 0 (0%) |

Table S2. Missing data analysis with regards to gender and the survey question “How important is it for you to have access to the following information which is wholly or partly based on information contained in “Journalen”.

|  | **Women** | **Men** | **Other** |
| --- | --- | --- | --- |
| **Referrals (content and how it is handled in care)**, n(%) | 31 (1.9%) | 18 (2.3%) | 0 (0%) |
| **List of all pharmaceuticals**, n(%) | 28 (1.7%) | 15 (1.9%) | 0 (0%) |
| **Overview of all vaccinations**, n(%) | 35 (2.1%) | 26 (3.3%) | 0 (0%) |
| **Results of tests**, n(%) | 26 (1.6%) | 14 (1.8%) | 0 (0%) |
| **Overview of all health care contacts**, n(%) | 39 (2.4%) | 20 (2.5%) | 0 (0%) |
| **Ability to order and manage medical certificate and other certificates**, n(%) | 43 (2.6%) | 31 (3.9%) | 0 (0%) |
| **Ability to point out errors I find in the record**, n(%) | 48 (2.9%) | 30 (3.8%) | 0 (0%) |
| **Ability to write comments to text in “Journalen”**, n(%) | 44 (2.7%) | 29 (3.6%) | 0 (0%) |
| **Contribute with information on health, for example by providing Health declaration for next visit**, n(%) | 46 (2.8%) | 28 (3.5%) | 0 (0%) |
| **Contribute with information of self-testing/monitoring at home**, n(%) | 56 (3.4%) | 28 (3.5%) | 1 (10%) |
| **Contribute information about expectations for the health care visit**, n(%) | 59 (3.6%) | 31 (3.9%) | 0 (0%) |
| **Ability to contact health care electronically and ask questions about medical record**, n(%) | 50 (3.1%) | 26 (3.3%) | 0 (0%) |
| **Ability to block certain medical records from access by other medical staff**, n(%) | 55 (3.4%) | 36 (4.5%) | 0 (0%) |
| **See which care units and staff groups have been inside “Journalen” (see log data)**, n(%) | 44 (2.7%) | 35 (4.4%) | 0 (0%) |
| **Ability to access information and manage services for my children**, n(%) | 96 (5.9%) | 60 (7.5%) | 0 (0%) |

Table S3. Missing data analysis with regards to education and survey questions about communication with medical staff and reasons for use.

|  | **No formal** | **Primary** | **Seconday** | **Higher** | **Research** |
| --- | --- | --- | --- | --- | --- |
| **Information in Journalen has helped me in communication with medical staff**, n(%) | 2 (3.0%) | 7 (4.4%) | 16 (2.4%) | 38 (2.7%) | 2 (2.7%) |
| **I read my health record online mostly out of general interest**, n(%) | 3 (4.5%) | 12 (7.5%) | 33 (5.0%) | 71 (5.0%) | 3 (4.0%) |
| **I read my health record online to get an overview of my medical history and treatment**, n(%) | 3 (4.5%) | 4 (2.5%) | 13 (2.0%) | 29 (2.1%) | 0 (0%) |
| **I read my health record online because I am not sure if I got the right care**, n(%) | 2 (3.0%) | 17 (10.7%) | 30 (4.6%) | 68 (4.8%) | 4 (5.3%) |
| **I read my health record online because I suspect inaccuracies**, n(%) | 2 (3.0) | 16 (10.1%) | 30 (4.6%) | 66 (4.7%) | 4 (5.3%) |
| **I read my health record online to prepare for my health care visit**, n(%) | 3 (4.5%) | 14 (8.8%) | 23 (3.5%) | 48 (3.4%) | 3 (4.0%) |

Table S4. Missing data analysis with regards to education and the survey question “How important is it for you to have access to the following information which is wholly or partly based on information contained in “Journalen”.

|  | **No formal** | **Primary** | **Secondary** | **Higher** | **Research** |
| --- | --- | --- | --- | --- | --- |
| **Referrals (content and how it is handled in care)**, n(%) | 1 (1.5%) | 5 (3.1%) | 12 (1.8%) | 26 (1.8%) | 1 (1.3%) |
| **List of all pharmaceuticals**, n(%) | 3 (4.5%) | 6 (3.8%) | 7 (1.1%) | 25 (1.8%) | 1 (1.3%) |
| **Overview of all vaccinations**, n(%) | 2 (3.0%) | 8 (5.0%) | 15 (2.3%) | 30 (2.1%) | 1 (1.3%) |
| **Results of tests**, n(%) | 3 (4.5%) | 7 (4.4%) | 9 (1.4%) | 19 (1.3%) | 1 (1.3%) |
| **Overview of all health care contacts**, n(%) | 0 (0%) | 8 (5.0%) | 15 (2.3%) | 30 (2.1%) | 2 (2.7%) |
| **Ability to order and manage medical certificate and other certificates**, n(%) | 2 (3.0) | 11 (6.9%) | 15 (2.3%) | 39 (2.8%) | 1 (1.3%) |
| **Ability to point out errors I find in the record**, n(%) | 1 (1.5%) | 13 (8.2%) | 23 (3.5%) | 36 (2.6%) | 2 (2.7%) |
| **Ability to write comments to text in “Journalen”**, n(%) | 1 (1.5%) | 10 (6.3%) | 19 (2.9%) | 37 (2.6%) | 1 (1.3%) |
| **Contribute with information on health, for example by providing Health declaration for next visit**, n(%) | 3 (4.5%) | 12 (7.5%) | 18 (2.7%) | 35 (2.5%) | 1 (1.3%) |
| **Contribute with information of self-testing/monitoring at home**, n(%) | 2 (3.0%) | 9 (5.7%) | 19 (2.9%) | 46 (3.3%) | 2 (2.7%) |
| **Contribute information about expectations for the health care visit**, n(%) | 2 (3.0) | 11 (6.9%) | 22 (3.4%) | 43 (3.0%) | 4 (5.3%) |
| **Ability to contact health care electronically and ask questions about medical record**, n(%) | 2 (3.0%) | 12 (7.5%) | 17 (2.6%) | 38 (2.7%) | 3 (4.0%) |
| **Ability to block certain medical records from access by other medical staff**, n(%) | 1 (1.5%) | 12 (7.5%) | 23 (3.5%) | 43 (3.0%) | 2 (2.7%) |
| **See which care units and staff groups have been inside “Journalen” (see log data)**, n(%) | 1 (1.5%) | 12 (7.5%) | 20 (3.0%) | 37 (2.6%) | 1 (1.3%) |
| **Ability to access information and manage services for my children**, n(%) | 4 (6.0%) | 21 (13.2%) | 32 (4.9%) | 83 (5.9%) | 4 (5.3%) |

Table S5. Missing data analysis with regards to age and survey questions about communication with medical staff and reasons for use.

|  | **15-24** | **25-44** | **45-64** | **65-84** | **> 85** |
| --- | --- | --- | --- | --- | --- |
| **Information in Journalen has helped me in communication with medical staff**, n(%) | 1 (0.7%) | 14 (1.8%) | 19 (2.0%) | 37 (6.4%) | 1 (4.2%) |
| **I read my health record online mostly out of general interest**, n(%) | 1 (0.7%) | 14 (1.8%) | 49 (5.2%) | 67 (11.6%) | 1 (4.2%) |
| **I read my health record online to get an overview of my medical history and treatment**, n(%) | 2 (1.4%) | 10 (1.3) | 16 (1.7%) | 19 (3.3%) | 2 (8.3%) |
| **I read my health record online because I am not sure if I got the right care**, n(%) | 3 (2.1%) | 15 (2.0%) | 41 (4.4%) | 71 (12.2%) | 1 (4.2%) |
| **I read my health record online because I suspect inaccuracies**, n(%) | 2 (1.4%) | 18 (2.4%) | 38 (4.0%) | 68 (11.7%) | 1 (4.2%) |
| **I read my health record online to prepare for my health care visit**, n(%) | 1 (0.7%) | 12 (1.6%) | 34 (3.6%) | 51 (8.8%) | 2 (8.3%) |

Table S6. Missing data analysis with regards to age and the survey question “How important is it for you to have access to the following information which is wholly or partly based on information contained in “Journalen”.

|  | **15-24** | **25-44** | **45-64** | **65-84** | **> 85** |
| --- | --- | --- | --- | --- | --- |
| **Referrals (content and how it is handled in care)**, n(%) | 1 (0.7%) | 6 (0.8%) | 19 (2.0%) | 21 (3.6%) | 2 (8.3%) |
| **List of all pharmaceuticals**, n(%) | 0 (0%) | 7 (0.9%) | 16 (1.7%) | 19 (3.3%) | 0 (0%) |
| **Overview of all vaccinations**, n(%) | 0 (0%) | 7 (0.9%) | 21 (2.2%) | 31 (5.3%) | 1 (4.2%) |
| **Results of tests**, n(%) | 0 (0%) | 11 (1.4) | 11 (1.2%) | 16 (2.8%) | 1 (4.2%) |
| **Overview of all health care contacts**, n(%) | 0 (0%) | 10 (1.3%) | 22 (2.3%) | 25 (4.3%) | 1 (4.2%) |
| **Ability to order and manage medical certificate and other certificates**, n(%) | 1 (0.7%) | 9 (1.2%) | 16 (1.7%) | 46 (7.9%) | 1 (4.2%) |
| **Ability to point out errors I find in the record**, n(%) | 0 (0%) | 11 (1.4%) | 26 (2.8%) | 39 (6.7%) | 1 (4.2%) |
| **Ability to write comments to text in “Journalen”**, n(%) | 1 (0.7%) | 10 (1.3%) | 20 (2.1%) | 40 (6.9%) | 1 (4.2%) |
| **Contribute with information on health, for example by providing Health declaration for next visit**, n(%) | 1 (0.7%) | 10 (1.3%) | 21 (2.2%) | 40 (6.9%) | 1 (4.2%) |
| **Contribute with information of self-testing/monitoring at home**, n(%) | 1 (0.7%) | 9 (1.2%) | 29 (3.1%) | 43 (7.4%) | 1 (4.2%) |
| **Contribute information about expectations for the health care visit**, n(%) | 2 (1.4%) | 14 (1.8%) | 28 (3.0%) | 44 (7.6%) | 1 (4.2%) |
| **Ability to contact health care electronically and ask questions about medical record**, n(%) | 2 (1.4%) | 11 (1.4%) | 23 (2.4%) | 39 (6.7%) | 1 (4.2%) |
| **Ability to block certain medical records from access by other medical staff**, n(%) | 3 (2.1%) | 12 (1.6%) | 31 (3.3%) | 43 (7.4%) | 1 (4.2%) |
| **See which care units and staff groups have been inside “Journalen” (see log data)**, n(%) | 1 (0.7%) | 10 (1.3%) | 26 (2.8%) | 40 (6.9%) | 1 (4.2%) |
| **Ability to access information and manage services for my children**, n(%) | 0 (0%) | 13 (1.7%) | 52 (5.5%) | 91 (15.7%) | 1 (4.2%) |
